# Perceived Academic Control and Academic Emotions Predict Undergraduate University Student Success: Examining Effects on Dropout Intention and Achievement

**DOI:** 10.3389/fpsyg.2017.00243

**Published:** 2017-03-07

**Authors:** Lisa Respondek, Tina Seufert, Robert Stupnisky, Ulrike E. Nett

**Affiliations:** ^1^Institute of Psychology and Education, Ulm UniversityUlm, Germany; ^2^Educational Foundations and Research, University of North DakotaGrand Forks, ND, USA; ^3^Empirical Educational Research, University of AugsburgAugsburg, Germany

**Keywords:** perceived control, academic emotion, freshman, academic success, dropout intention, academic achievement, higher education, multi-group structural equation modeling

## Abstract

The present study addressed concerns over the high risk of university students' academic failure. It examined how perceived academic control and academic emotions predict undergraduate students' academic success, conceptualized as both low dropout intention and high achievement (indicated by GPA). A cross-sectional survey was administered to 883 undergraduate students across all disciplines of a German STEM orientated university. The study additionally compared freshman students (*N* = 597) vs. second-year students (*N* = 286). Using structural equation modeling, for the overall sample of undergraduate students we found that perceived academic control positively predicted enjoyment and achievement, as well as negatively predicted boredom and anxiety. The prediction of dropout intention by perceived academic control was fully mediated via anxiety. When taking perceived academic control into account, we found no specific impact of enjoyment or boredom on the intention to dropout and no specific impact of all three academic emotions on achievement. The multi-group analysis showed, however, that perceived academic control, enjoyment, and boredom among second-year students had a direct relationship with dropout intention. A major contribution of the present study was demonstrating the important roles of perceived academic control and anxiety in undergraduate students' academic success. Concerning corresponding institutional support and future research, the results suggested distinguishing incoming from advanced undergraduate students.

## Predictors of undergraduate university students' academic success

Many studies have found that freshman university students are at high risk of attrition [NCES (National Center for Education Statistics), 2002; AUSSE (Australasian Survey of Student Engagement), 2011]. These students often face difficulties in the transition to higher education and experience varying degrees of adjustment to university during the first year, which in turn predict their academic success (Credé and Niehorster, 2012). The first academic year is critical to the overall success in higher education (Perry et al., 2001). In order to successfully manage this critical adjustment to university, one key factor to consider is the undergraduate students' feeling “in control” over their academic outcomes (Perry, 1991). Additionally, the new demands and rising academic pressure of university are likely to elicit a variety of emotions among students, which can influence their academic success (Pekrun and Stephens, 2010). Understanding the role of these variables for undergraduate students' academic success, specifically with respect to lowering dropout and increasing achievement, is a key factor for instructors, professors, and institutions in order to support student development. However, few studies have focused on perceived academic control as a predictor of dropout intention, in addition to its influence on achievement, even though instructors and institutions can effectively support it. Moreover, few studies have examine academic emotions as a predictor for dropout intention, in addition to their influence on achievement. The purpose of the present study therefore was to examine how perceived academic control and academic emotions predict undergraduate university students' academic success. The next section describes the theoretical background of the constructs under investigation, with a focus on their relationship with academic success and the undergraduate university experience.

### Undergraduate students' academic success

In the research literature, two components are prevalently discussed as representing undergraduate students' academic success: dropout intention and academic achievement. Engagement in higher education is typically elective and students need to remain enrolled to stay on track. Unfortunately, dropout rates within higher education worldwide suggest approximately one-third of university students leave university in their first year (OECD (Organization for Economic Co-operation and Development), 2012). Dropping out is often seen as an individual failure with negative societal consequences, such as fewer qualified employees in the workforce (Heublein and Wolter, 2011). The European Commission is focusing on reducing dropout rates by encouraging research on this issue (DG EAC (Directorate General for Education and Culture, European Commission), 2015). Research concerning dropout faces the problems of tracking students when they leave institutions or stop studying (Pascarella and Terenzini, 2005). Therefore, prior research has focused mainly on cognate constructs, such as retention, persistence (defined by the length of time a student remains enrolled at an institution; for an overview see Robbins et al., 2004), or voluntary course withdrawal (Ruthig et al., 2004).

Based on the difficulties in measuring and testing factors affecting dropouts that have already occurred, it is essential to know which students *intend to drop out* before they actually do. Bean (1982) analyzed dropout intention, the estimated likelihood of suspending studies, as part of dropout syndrome within his Student Attrition Model. The relationship between dropout intention and actual dropout has been consistently emphasized (cf. Cabrera et al., 1993). Prior research studied possible moderators, such as commitment and engagement (cf. Okun et al., 1996) or possible institutional interventions, such as freshmen seminars for study skills or social integration (Porter and Swing, 2006; DeAngelo, 2014). Bean highlighted the importance of dropout intention: “Students who leave without intending to (e.g., for reasons of health, family crisis, etc.) do not represent failures of the student or the university. They represent residual variance in dropout that can be accurately specified after the fact, but not predicted.” (Bean, 1985, p. 36). Dropout intention among undergraduate students seems to decrease in the second academic year compared to the first year (Bean, 1985). Consequently, the current study focused on students' dropout intention, operationalized as students' reported intent to change their major or leave their university, as an early-warning sign of actual dropout (Bean, 1985).

In addition to low dropout intention, prior research traditionally defined academic success as achievement based on course grades or grade point average (GPA; Richardson et al., 2012). University *academic achievement* has been found to predict educational and career success. Meta-analysis demonstrated that grades are positively related to career success, besides intelligence or parental socioeconomic status (Strenze, 2007). For example, course achievement at the very beginning of the first academic year predicted final course grades for the rest of the first year (Perry et al., 2001).

We thus conceptualized academic success as built out of two components: (a) low dropout intention (as a predictor of dropout) and (b) high academic achievement (GPA). Prior research has shown retention (i.e., no dropout) and academic achievement are related to each other (e.g., Robbins et al., 2004, 2009; Gramling, 2013). Furthermore, most studies found a medium relationship between dropout and academic achievement as undergraduate students who achieve high grades are less likely to drop out (e.g., Pascarella and Terenzini, 2005; Allen et al., 2008). Focusing on all undergraduate students there is evidence that dropout intention is significantly related to low academic achievement, although with small effect sizes and without considering the duration of study (Bean, 1985). On the one hand, high achievement will convince students they have made the right choice to enroll in university and hence will reduce the urge to leave. On the other hand, the relationship might work the other way round: Students with low dropout intention might have the feeling of being at the right place, will be able to focus on their studies, and therefore may show high performance. The reducing impact of achievement on dropout intention was shown for high school grades (Porter and Swing, 2006). Allen et al. (2008) suggested that low grades more strongly relate to dropout decisions in the first academic year than later on (similar to Bean, 1985). Thus, the relationship between dropout intention and academic achievement reduces during longer periods of study. Further research is needed on the correlation between these two variables, specifically for freshman students.

It is clear that the first year of university is a critical time for students' early and long-term academic success (Credé and Niehorster, 2012). The answers are less clear, however, for the question of what factors influence academic success. Therefore, in the present study we explored the impact of perceived academic control and academic emotions on (a) dropout intention and (b) academic achievement.

### Perceived academic control

Perceived academic control has been found to be an important predictor of academic success in terms of (a) low dropout intention and (b) high achievement (for an overview see Perry et al., 2005b). Perceived control is often described as the subjective perception of individual influence; in other words, being in control (Skinner, 1996). Perceived academic control, the domain specific variant of perceived control, is a person's belief in his or her influence over the success or failure of achievement outcomes. It describes the personal internal attribution of achievement outcomes and is a relatively stable psychological disposition with state qualities (Perry et al., 2001, 2005a). Stupnisky et al. (2012) found perceived academic control to be unstable for some individuals and that this instability can have important consequences for their academic achievement. Perceived academic control is positively linked to several relevant factors, which underlines its importance for undergraduate students' academic success (for an overview, see Skinner, 1996; Perry et al., 2005a). Perceived academic control is closely related to self-efficacy (Judge et al., 2002), as both constructs are part of the expectancy component of students' self-concept (Pintrich and de Groot, 1990). Moreover, perceived academic control has a higher impact on academic success than self-esteem (Stupnisky et al., 2007). Other factors positively related to perceived control are self-regulated learning (Shell and Husman, 2008), effective study strategies use (Cassidy and Eachus, 2000), self-monitoring strategies use (Perry et al., 2001), achievement motivation (Hall et al., 2006), intrinsic motivation (Perry et al., 2001), and personality constructs such as extraversion or conscientiousness (Perry et al., 2005a).

Perceived academic control is also very important for undergraduate students during the challenging transition from secondary school to university. Entry into university means greater academic demands, but also greater autonomy, less academic structure, increased pressure to excel, new social environments, and adaption to new roles or responsibilities. These new demands foster a low-control learning environment in university and can lead students to feel out of control (Perry, 2003). Stupnisky et al. (2012) found that freshman students' academic control levels can be unstable (i.e., fluctuating between high and low) and often decrease within their first year.

Concerning *dropout*, there is evidence that students with high levels of perceived academic control are less likely to dropout (Perry et al., 2005b) and withdraw from courses (Ruthig et al., 2007), specifically within the first academic year (Hall et al., 2006). Similar results were found for secondary school students (Rumberger and Lim, 2008). Perceived academic control is furthermore related to psychology freshman students' *intention to drop out* of university (Ruthig's AERA presentation 2002 as cited in Perry et al., 2005a, p. 384) and students' intention to drop out in high school (Davis et al., 2002).

Within the academic context, perceived control has been linked to several components of university students' *academic achievement* (for an overview see Perry et al., 2005b). Prior research found perceived academic control to positively predict students' achievement over an entire first academic year (e.g., Ruthig et al., 2008; Daniels et al., 2014). Remarkably, perceived academic control significantly predicted academic achievement even after considering students' pre-university academic success, age, and gender (Stupnisky et al., 2007). Freshmen with high unstable perceived academic control were found to have lower academic achievement than high stable perceived academic control students, with low-unstable perceived academic control students to have the worst academic achievement (Stupnisky et al., 2012). Moreover, perceived academic control negatively predicted course-withdrawal and positively predicted achievement over a 3-year period (Perry et al., 2005b).

Due to its crucial role in the difficult transition to university, prior research has focused on the enhancement of perceived academic control in low-control students through Attributional Retraining (Perry et al., 2005a; for an overview of this intervention see Haynes et al., 2009). In order to improve perceived academic control, this teaching method encourages students to reflect on past performances and make controllable, unstable attributions for negative academic experiences. Attributional Retraining early in the academic year has been shown to result in better performance (Perry et al., 2005a).

In summary, the research literature suggests perceived academic control is an important predictor of undergraduate university student academic success for both (a) dropout intention and (b) academic achievement. Furthermore, perceived control can be increased by institutions to assist students. Thus, perceived academic control “provides students with the resources to overcome various educational obstacles” (Perry et al., 2005a, p. 423). In addition to perceived control, the high demands and rising academic pressure of university are likely to raise a variety of emotions among students, which then may also predict students' academic success (Pekrun and Stephens, 2010).

### Academic emotions

Academic emotions are those emotions relating to achievement activities, such as studying at university and test results (Pekrun, 2006). Discrete emotions such as enjoyment, boredom, and anxiety can be distinguished from general affect and are experienced in different frequencies. The most reported emotion within the higher education context is anxiety (Pekrun and Stephens, 2010), while enjoyment and boredom are also frequently reported (Pekrun et al., 2002). These discrete emotions relate to each other (Pekrun et al., 2011) and can change over time. Ranellucci et al. (2015) found that undergraduate students self-reported slightly less enjoyment, less anxiety, and nearly the same experience of boredom during their second academic year compared to their first. Alternatively, Pekrun et al. (2014) reported increasing boredom levels for undergraduate students over one semester. Misra and McKean (2000) showed a negative trend between self-reported anxiety levels for freshmen and second-year students, however it was not significant (_Δ_*M* = −0.20, *p* = 0.21).

Concerning *dropout*, there is evidence that negative academic emotions relate to voluntary course withdrawal, while positive academic emotions do not (e.g., Ruthig et al., 2004, 2007, 2008). Moreover, negative emotions tend to be higher in students who dropped out than in students who completed their studies (Ruthig's AERA 2002 presentation as well as Ziegler's master thesis 2001 as cited in Pekrun et al., 2002). In these studies, anxiety is frequently reported (Pekrun and Stephens, 2010).

Concerning *academic achievement*, previous research revealed that positive academic emotions (e.g., enjoyment) are positively related to future academic achievement, whereas negative academic emotions (e.g., boredom or anxiety) are negatively related (e.g., Pekrun and Stephens, 2010). The predictive effects of academic emotions on achievement seem to be mediated by motivation, learning strategies, and self-regulation (e.g., Pekrun et al., 2011; Putwain et al., 2013; Mega et al., 2014). Furthermore, the relationship between academic emotions and academic achievement might be reciprocal (cf. Pekrun et al., 2014, 2017); that is, emotions can predict future achievement and can be predicted by prior achievement through success or failure experiences. Prior research found that high academic achievement at the beginning of the first academic year predicts positive academic emotions, which then lead to high achievement at the end of the first academic year (Putwain et al., 2013). Overall, these prior findings lead to the question, how do perceived academic control and academic emotions (specifically negative) together predict undergraduate students' academic success?

### Relationships between these predictors and undergraduate students' academic success

Prior research revealed that perceived academic control and academic emotions are both important predictors of academic success and are strongly interrelated (e.g., Perry et al., 2001; Ruthig et al., 2007; Pekrun et al., 2011). The feeling of being in control is positively associated with positive emotions such as enjoyment, and negatively related to negative emotions such as anxiety (Perry et al., 2001; Pekrun et al., 2004, 2011; Hall et al., 2006).

According to Pekrun (2006), appraisals of perceived academic control and value determine academic achievement directly, but also indirectly via their prediction of academic emotions. Hence, Pekrun's (2006) Control-Value Theory of Emotions offers an explanation of the structural pathways and possible indirect effects between these predictors for undergraduate students' academic success. In the current study, we only investigated the theoretical assumptions concerning perceived academic control as a first step due to its high importance for freshman university students (Perry, 2003), because it differs among undergraduate university students (Stupnisky et al., 2012), and it can be supported through university interventions (e.g., Pekrun, 2006). Subjective value otherwise is relatively high among freshmen as they just chose their major and difficult to increase through institutional activities (Dresel and Grassinger, 2013).

Focusing on perceived academic control's relationship with emotions, low perceived academic control has been found to predict anxiety, whereas high perceived academic control leads to enjoyment (Stupnisky et al., 2013). The relation between boredom and perceived control is assumed to be U-shaped (curvilinear), with extreme high and extreme low control conditions eliciting boredom among students (Pekrun et al., 2014). Focusing on freshman students, however, the demanding circumstances of university make “it likely that the high levels of perceived control that would promote boredom are rarely achieved in these environments” (Pekrun et al., 2014, p. 699). Indeed, prior research found a negative linear relationship between perceived academic control and undergraduate university students' boredom (Pekrun et al., 2010). Moreover, researchers have suggested that the effects of perceived academic control on both components of future academic success are partially mediated by academic emotions. For example, students who feel in control typically experience more emotions that are positive, and therefore are more likely to succeed in their studies (Goetz et al., 2010).

The relationships between perceived academic control and academic emotions in undergraduates are assumed equal across different periods of study (Pekrun, 2006). The mean levels of these variables could differ, however, based on culture (Frenzel et al., 2007) or students' changing frame of reference. For example, freshman students are expected to relate university experiences to those from secondary school, whereas second-year students to those from higher education experiences. As second-year students have adapted to university and gained experience, they should report more university related self-perceptions.

In summary, based on the control-value theory (Pekrun, 2006), we hypothesized both perceived academic control and academic emotions to predict (a) dropout intention and (b) academic achievement. Moreover, we expected that the predictive effect of perceived academic control on success to be partially mediated by academic emotions.

## Study purpose and hypothesis

In the present study, we examined the impact of perceived academic control on the two academic success components, dropout intention and academic achievement, as well as possible mediations through academic emotions. We focused on university students in their first two academic years as this is a critical time for long-term academic success (e.g., Perry, 1991; Credé and Niehorster, 2012). We additionally explored differences amongst the relationships of our study variables for students at the first academic year (freshman group) compared to students at the second academic year (second-year group).

**Figure 1:**
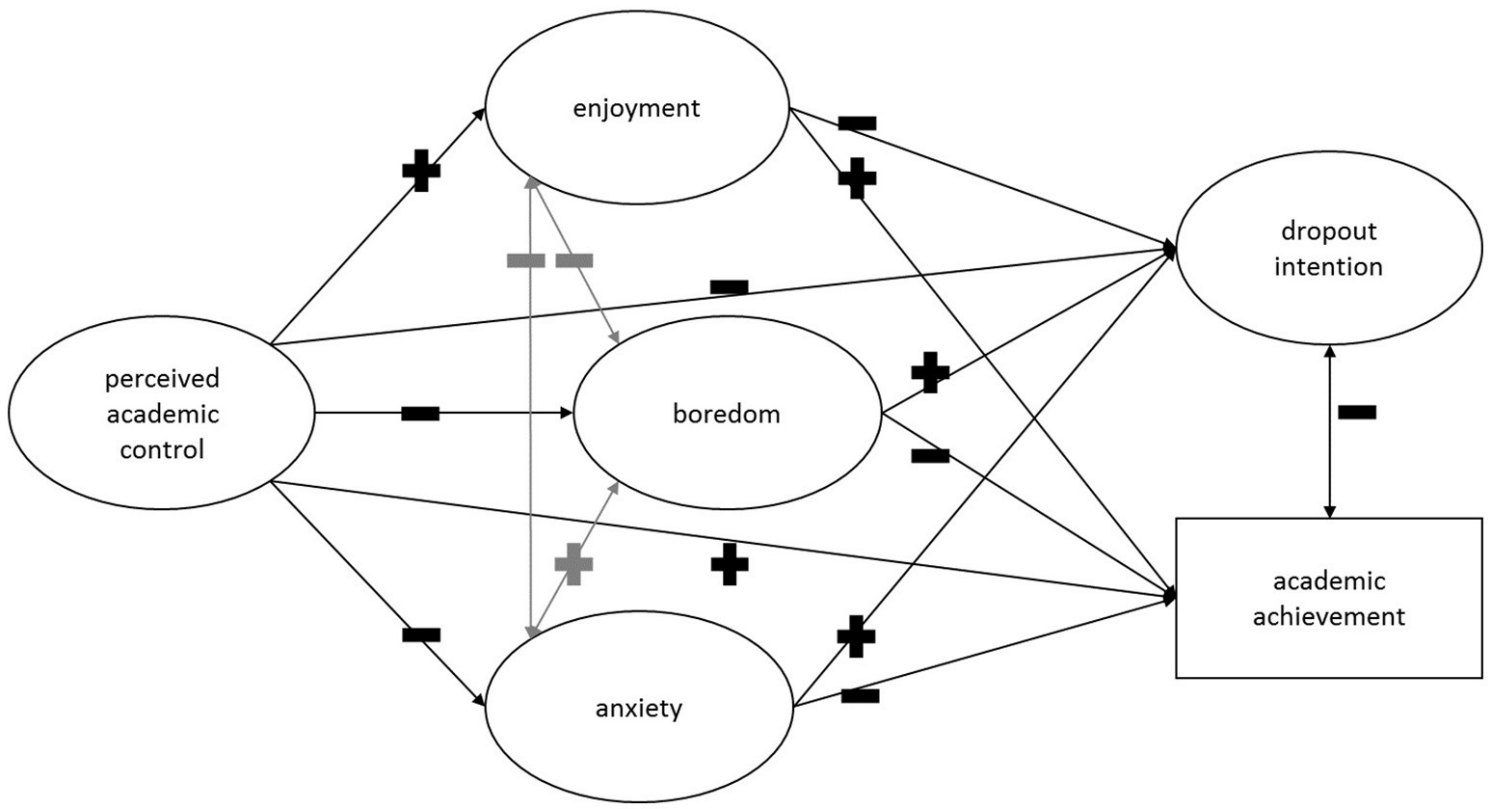
**Hypothesized model**.^1^

First, we analyzed the relationships of perceived academic control and academic emotions, as well as their connection with undergraduate students' academic success. We hypothesized a model inspired by the control-value theory of emotions (Pekrun, 2006) with a focus on perceived academic control appraisals and the addition of dropout intention (Figure 1). In our assumed model, perceived academic control had a direct effect on the two components of academic success by reducing dropout intention and enhancing academic achievement (c.f. Ruthig et al., 2008). Additionally, we hypothesized an indirect effect of perceived academic control on dropout intention and academic achievement through the academic emotions of enjoyment, boredom, and anxiety. Concerning the academic emotions, we expected them to correlate with one another. Specifically, we expected negative relationships between enjoyment and the negative emotions of boredom and anxiety, while we expected the negative emotions to be positively related to each other (Pekrun et al., 2011). Furthermore, we hypothesized that enjoyment relates to great undergraduate students' academic success (c.f. Ruthig et al., 2008). However, we hypothesized a strong negative relationship of boredom and anxiety with academic success (c.f. Stupnisky et al., 2013) due to the high frequency and educational importance (Pekrun and Stephens, 2010). We additionally assumed a correlation between the two components of academic success for undergraduate university students (Bean, 1985; Pascarella and Terenzini, 2005; Allen et al., 2008).

Second, we wanted to compare students of different durations of study within the first two academic years. We tested our model (Figure 1) separately for freshmen and second-year students. Based on Pekrun's (2006) theory, we expected to find our model structurally invariant across the two student groups of freshmen and second-year students. However, we hypothesized slightly different mean levels depending on the students' year in university. Specifically, we expected to find less perceived academic control, more enjoyment, less anxiety, less dropout intention, and higher academic achievement for second-year students compared to freshmen based on previous research (Bean, 1985; Perry et al., 2005b; Stupnisky et al., 2012).

## Materials and methods

### Participants

Participants were 883 undergraduate students (48.4% women), whose mean age was 20.23 years with a standard deviation of 2.54 (range from 16 to 50 years). They were studying across all disciplines offered by a German university with focus on STEM (engineering, computer science, mathematics and economics, psychology as well as physics, biology, chemistry). The German academic year is comprised of two semesters with an exam period at the end of each semester. Freshman students normally experience their first exam in February. This cross-sectional study included participants from two different cohorts in order to test the multi-group hypothesis: 597 first-year students (freshman group, 41.6% response rate of total cohort number one) and 286 second-year students (second-year group, 19.2% response rate of total cohort number two).

The study was conducted in two phases. Participants were recruited about 2–3 weeks into the academic year when students were either at the beginning of their first semester or third semester (Phase 1–survey, November). Thus, we collected data from students at the beginning of their program when they had no exam experience (freshman student cohort—group one), as well as from students at the beginning of their second academic year, when they had just finished their first academic year (second-year student cohort—group two). We distributed the questionnaire during a break in an important lecture of each discipline. Therefore, this was a convenience sample and participants responded to the survey about experiences at university while being in a typical learning situation. Participation was voluntary and participants had the chance to withdraw their data at any point of the study. In addition to the survey, students gave permission to the authors to obtain the grade point average (GPA) by signing a data privacy statement (grade release form). After the conclusion of the semester, students' GPA was collected from institutional records (Phase 2—GPA collection, April). In the present study, we analyzed data only from students with available grade information (*N* = 883 of the original 1171 participants^2^, with 597 freshmen from original 790 participant and 286 second-year students from original 381 participants).

### Measures

All measures in the paper-pencil questionnaire were established self-report scales. When necessary, we adapted the items from a secondary to tertiary education context with a general focus on studying and lectures.

#### Perceived academic control

We adapted six items of the Academic Control Scale (PAC; Perry, 1991 in its German version of Pekrun et al., 2004) to the context of higher education, which were measured on a five-point Likert scale (1 = Strongly disagree; 5 = Strongly agree). An example item was, “The more effort I put into my courses, the better I do in them.”

#### Academic emotion

The discrete emotions of enjoyment, boredom, and anxiety were each measured with three course-related items from the German version of the Academic Emotions Questionnaire (AEQ; Pekrun et al., 2005, 2011) on a five-point Likert scale (1 = Strongly disagree; 5 = Strongly agree). We selected these emotions due to their educational importance (Ruthig et al., 2008) as well as high frequency among higher education students (Pekrun et al., 2002). Example items were for enjoyment “I get excited about going to university,” for boredom “I think the courses of my study are boring,” and for anxiety “Thinking about my study makes me feel uneasy.”

#### Dropout intention

We adapted three items from the institutional commitment scale of the Freshman Orientation Survey (Brown, 2012). Students reported the likelihood of changing their major or leave their university (1 = Extremely unlikely; 4 = Extremely likely). An example item was “I am likely to leave university permanently.”

#### Academic achievement

Similar to prior research, academic achievement was operationalized through students' cumulative grade point averages (GPA). Despite known difficulties with reliability and disciplinary differences, it is still the most common achievement measure (Robbins et al., 2004). GPA was obtained from institutional records at the end of the semester. It contains students' average grade achieved in all courses completed during their semester and ranges from 1.0 (excellent) to 4.0 (passed). In Germany, lower grades represent higher academic achievement. Because of the irregular standards and entrance requirements between the different disciplines, GPA was group centered regarding to students' major before being used in our analysis. We subtracted the group mean for each group of study subject, across the whole sample independent of their student status (freshmen vs. second-year students)^3^. For an easy interpretation, GPA was then multiplied by −1 in order to recode it. For the final analyzed variable, a high GPA reflects high academic achievement, whereas a low GPA reflects low academic achievement.

## Results

### Rationale for analyses

We tested our hypotheses with bivariate correlations and multi-group structural equation modeling (SEM). Perceived academic control, academic emotions, and dropout intention were latent variables, while academic achievement was an observed variable. First, we conducted bivariate correlations among all study variables to analyze the relationships. Second, we tested our hypothesized model for the whole sample through SEM, which allowed testing of the theoretical linkages as direct and indirect partial relations (Byrne, 2012). Third, we tested our multi-group hypothesis through multiple invariance tests.

In order to compare the SEM between the two subgroups (freshmen vs. second-year student cohort), we tested the measurement invariance and the structural invariance of the hypothesized model (as recommend by Byrne, 2012; Christ and Schlüter, 2012; Wang and Wang, 2012). We used a series of tests to explore measurement invariance of the hypothesized model (i.e., hierarchical set of measurement invariance tests). We systematically added more constraints and evaluated the cross-group comparability through chi-square difference tests (Brown, 2006). Specifically, we tested for configural measurement invariance through establishing a model with no constraints (model 1) after separately testing the two baseline models within the subgroups (model 0). Here, the hypothesized model of both subgroups was freely estimated and allowed all cross-group differences. Next we tested for weak measurement invariance (Meredith, 1993) through constraining all factor loadings to be equal (model 2). This model assumed no differences between the factorial structures of both subgroups. At least partial invariance is necessary to compare structural differences (Byrne, 2012). Finally, we tested strong measurement invariance (Meredith, 1993) by constraining all intercepts of the manifest variables to be equal (model 6). Again, at least partial invariance is necessary to compare latent means across groups (Christ and Schlüter, 2012). As significant chi-square difference tests and worse model fits suggested partial invariance, we developed a series of partial invariance models to examine the origin of the lack of invariance by systematically releasing the constraints (model 3, 4 and model 6, 7).

After our successful test for measurement invariance, we examined the structural invariance of the hypothesized model (as recommend by Byrne, 2012). After we knew which of the measurement models were group-invariant, we constrained these to be equal across the two groups while we tested the invariance of structural parameters (latent means and regression paths). Specifically, we systemically constrained single structural weights to be equal across both subgroups. Again, we tested the cross-group comparability through chi-square difference tests. As significant chi-square difference tests suggested the structural weights were non-invariant, we compared the different structural weights across both subgroups. Indirect effects, meaning moderated mediations, were tested through *z*-score difference tests. This allows for comparing indirect effects within SEM across groups (Muthén and Muthén, 1998–2012). After we estimated the values of the single direct effects in both groups, we quantified the indirect effect as the product of the respective direct effects for each group and then we compared these interactions via *z*-score difference tests (cf. Wang and Wang, 2012). A significant *z*-score test suggests a significant difference between the two mediations, meaning a significant moderation of student year at university. In addition to this multi-group analysis, we tested the differences in academic achievement through *t*-tests.

All analyses were executed using Mplus 7 (Muthén and Muthén, 1998–2012) and we considered various fit indices based on Hu and Bentler (1999). Adequate model fit was indicated through chi-square (χ^2^), root-mean-square error of approximation (RMSEA ≤ 0.05), comparative fit index (CFI ≥ 0.95) and standardized root mean square residual (SRMR ≤ 0.05). Syntaxes of the models are provided as supplementary materials (Data Sheet 1).

### Descriptive statistics

Table 1 displays the descriptive results. All study variables showed expected average mean levels for the overall sample as well as for both student subgroups separately. We used McDonalds Omega to verify the internal reliability of our scales, with only boredom having omegas slightly under 0.70. All estimates were calculated via maximum likelihood estimation with missing data^4^ assumed to be missing at random—MAR (Muthén and Muthén, 1998–2012). Skewness and graphical check for anxiety and dropout intention suggested non-normal distributions (Miles and Shevlin, 2001). Therefore, we used the MLR maximum likelihood estimator to handle missing data, which is robust to non-normality, instead of the full information maximum likelihood estimator ML (Muthén and Muthén, 1998–2012).

**Table 1:** **Descriptive results of all study variables**.

**Variable**	**No. Items**	**Range**	**Group**	***N***	***M***	***SD***	**ω**	**Skewness**	**λ standardized**
Perceived Academic Control	6	1–5	0	855	3.99	0.61	0.81	−0.87	0.64/0.66/0.56 0.59/0.72/0.72
			1	572	4.04	0.55	0.76	−0.68	0.57/0.61/0.51 0.52/0.67/0.69
			2	283	3.89	0.72	0.87	−0.86	0.72/0.77/0.61 0.68/0.78/0.76
**Academic emotion**									
Enjoyment	3	1–5	0	856	3.66	0.67	0.81	−0.65	0.79/0.81/0.71
			1	578	3.67	0.67	0.81	−0.63	0.79/0.82/0.72
			2	278	3.62	0.67	0.80	−0.68	0.79/0.79/0.69
Boredom	3	1–5	0	857	2.43	0.79	0.69	0.28	0.74/0.57/0.53
			1	582	2.38	0.78	0.68	0.27	0.73/0.58/0.55
			2	275	2.52	0.80	0.62	0.29	0.79/0.53/0.47
Anxiety	3	1–5	0	855	1.86	0.79	0.75	0.98	0.67/0.68/0.75
			1	581	1.93	0.78	0.72	0.83	0.63/0.64/0.73
			2	274	1.72	0.79	0.81	1.38	0.73/0.76/0.80
**Academic success**									
Dropout intention	3	1–4	0	870	1.62	0.53	0.74	0.66	0.65/0.66/0.80
			1	585	1.73	0.51	0.70	0.41	0.59/0.59/0.80
			2	285	1.38	0.50	0.77	1.57	0.69/0.72/0.77
Academic achievement	−	−1.52–1.69	0	811	0.00	0.64	−	−0.15	−
		−1.52–1.53	1	530	−0.11	0.67	−	−0.33	−
		−0.99–1.69	2	281	0.18	0.53	−	−0.10	−

*λThe numbers refer to standardized MLR maximum likelihood factor loading estimates of the confirmatory factor analyses. Group 0 refer to total sample (N = 883). Group 1 refer to freshman students (N = 597). Group 2 refer to second-year students (N = 286). All significant λ were significant at p < 0.001*.

Latent variables were tested with confirmatory factor analysis (CFA) in the total sample and the two student groups separately. The CFA for the total sample had a close to adequate model fit: $\chi_{\left(125\right)}^{2}$ = 437.67, *p* < 0.001, RMSEA = 0.05, CFI = 0.93, SRMR = 0.04. Focusing on the two student groups separately, we again confirmed our latent study variables, with a slightly lower fit for the second-year student group [freshman sample: $\chi_{\left(125\right)}^{2}$ = 291.35, *p* < 0.001, RMSEA = 0.05, CFI = 0.93, SRMR = 0.04; advanced sample: $\chi_{\left(125\right)}^{2}$ = 270.35, *p* < 0.001, RMSEA = 0.06, CFI = 0.92, SRMR = 0.06]. We then checked the standardized factor loadings of all latent variables, which were higher than 0.4 (ranged from 0.47 to 0.82) as recommended by Stevens (2009). Thus, all factor structures representing the corresponding latent variable had close to adequate fit to the data with significant factor loadings for the full sample as well as the two subgroups. The latent variables were thus used for all further analyses.

Furthermore, we calculated the bivariate Pearson product-moment correlations between the latent variables (enjoyment, boredom, anxiety, perceived academic control, dropout intention) and the observed variable achievement. In general, most bivariate correlations were consistent with prior research, and in some cases were even stronger than in previous research. Strong negative correlations were found between enjoyment and boredom, in all groups and the total sample. We found the following relationships in the complete sample of undergraduate students (see Table 2). As expected, perceived academic control related significantly to all three academic emotions, particularly to anxiety. Furthermore, the academic emotions strongly related to each other. Both perceived academic control and academic emotions were moderately to strongly related to dropout intention; however, anxiety had the strongest relationship to dropout intention. Concerning academic achievement, we generally found small correlations, whereas perceived academic control and dropout intention had the largest relations to GPA. The more students self-reported perceived academic control, as well as the less students self-reported boredom, anxiety, or dropout intention, the more likely they achieved highly in the end of the semester.

**Table 2:** **Bivariate correlations of all latent study variables (total sample)**.

**Variable**	**1**	**2**	**3**	**4**	**5**	**6**
**Self-concept**						
1. Perceived academic control		0.30^***^	−0.39^***^	−0.52^***^	−0.34^***^	0.17^***^
**Academic emotion**						
2. Enjoyment			−0.80^***^	−0.54^***^	−0.42^***^	0.06
3. Boredom				0.43^***^	0.26^***^	−0.14^**^
4. Anxiety					0.64^***^	−0.11^**^
**Academic success**						
5. Dropout intention						−0.18^***^
6. Academic achievement						

*The numbers refer to standardized MLR maximum likelihood parameter estimates*.

** *p < 0.01*,

*** *p < 0.001*.

When the student groups were compared (see Table 3), we found a similar pattern of correlations. One exception was that achievement had very different relationships with all other latent variables across the groups. For freshman students, GPA relations were weak or non-significant with other variables, while second-year students showed GPA to have moderate relations with all latent study variables, except for enjoyment. The more second-year students self-reported perceived academic control, and the less they reported boredom, anxiety, or dropout intention, the more likely they achieved highly at the end of the third semester. In addition to these correlations we performed SEM to isolate the specific relationships of our two key study variables from those of other study variables and reduce the measurement error through latent variables.

**Table 3:** **Bivariate correlations of all latent study variables (separate subgroup samples)**.

**Variable**	**1**	**2**	**3**	**4**	**5**	**6**
**Self-concept**						
1. Perceived academic control		0.31^***^	−0.39^***^	−0.55^***^	−0.33^***^	0.13^**^
**Academic Emotion**						
2. Enjoyment	0.28^***^		−0.74^***^	−0.56^***^	−0.47^***^	0.10^**^
3. Boredom	−0.39^***^	−0.92^***^		0.42^***^	0.31^***^	−0.16^**^
4. Anxiety	−0.55^***^	−0.52^***^	0.49^***^		0.62^***^	−0.05
**Academic success**						
5. Dropout intention	−0.52^***^	−0.42^***^	0.30^**^	0.69^***^		−0.06
6. Academic achievement	0.34^***^	0.00	−0.18^**^	−0.19^**^	−0.25^**^	

*The numbers refer to standardized MLR maximum likelihood parameter estimates. The numbers above the diagonal refer to freshman students. The numbers under the diagonal refer to second-year students*.

** *p < 0.01*,

*** *p < 0.001*.

### Research question 1–testing the relationships between perceived academic control and academic emotions and their effect on undergraduate students' academic success

We next tested our hypothesized model for the total student data through SEM. Table 4 (first column) displays the results of the SEM. This initial total sample SEM analysis showed an acceptable model fit [$\chi_{\left(138\right)}^{2}$ = 489.58, *p* < 0.001, RMSEA = 0.05, CFI = 0.92, SRMR = 0.05]. Based on *post-hoc* analysis, we allowed measurement errors to correlate, specifically on both boredom and anxiety scales' two items, due to high measurement residual covariance cross-loadings and high expected parameter change (EPC) values (boredom: MI = 80.52, EPC = 0.33; anxiety MI = 32.21, EPC = 0.16). Byrne (2012) argued that high MI and EPC values represent necessary model specification due to systematic measurement errors in item responses from a high degree of overlap in the item content (e.g., for boredom “When I think about class, I get queasy” and “Thinking about class makes me feel uneasy”). After modification, the model showed a better and adequate model fit: χ^2^_(136)_ = 377.02, *p* < 0.001, RMSEA = 0.04, CFI = 0.95, SRMR = 0.04.

**Table 4:** **Direct and indirect effects on academic success**.

**Direct relation**	**Hypothesized model**			**Final partial invariant multi-group model**					
	**Total sample (*N* = 883)**			**Freshman sample (*N* = 597)**			**Second-year sample (*N* = 286)**		
	***b*/*r* (*p*)**	**β/*r_*stdxy*_* (*p*)**	**R^2^**	***b*/*r* (*p*)**	**β/*r_*stdxy*_* (*p*)**	**R^2^**	***b*/*r* (*p*)**	**β/*r_*stdxy*_* (*p*)**	**R^2^**
Dropout Intention			0.48^***^			0.49^***^			0.52^***^
Perceived academic control	0.00 (0.946)	0.00 (0.946)		0.08 (0.569)	0.06 (0.564)		−0.26^*^	−0.18^†^	
Enjoyment	−0.15 (0.102)	−0.21 (0.102)		−0.18 (0.385)	−0.14 (0.392)		−0.91^*^	−0.66^*^	
Boredom	−0.12 (0.162)	−0.18 (0.159)		−0.03 (0.883)	−0.02 (0.883)		−0.89^*^	−0.64^*^	
Anxiety	0.47^***^	0.64^***^		0.75^***^	0.66^***^		0.66^***^	0.54^***^	
Academic achievement	−0.25^*^	−0.17^*^		−0.01 (0.737)	−0.02 (0.737)		−0.08 (0.133)	−0.17(0.141)	
Academic Achievement			0.04^*^			0.03 (0.139)			0.16^*^
Perceived academic control	0.20^**^	0.16^**^		0.12^*^	0.18^*^		0.17^***^	0.33^***^	
Enjoyment	−0.05 (0.741)	−0.05 (0.741)		0.09 (0.306)	0.14 (0.306)		−0.31 (0.386)	−0.62 (0.386)	
Boredom	−0.02 (0.893)	−0.02 (0.893)		0.03 (0.725)	0.05 (0.724)		−0.24 (0.504)	−0.47 (0.503)	
Anxiety	0.06 (0.514)	0.06 (0.514)		0.06 (0.317)	0.11 (0.319)		−0.05 (0.371)	−0.11 (0.366)	
Enjoyment			0.10^**^			0.11^**^			0.07^†^
Perceived academic control	0.37^***^	0.31^***^		0.35^***^	0.33^***^		0.27^***^	0.27^***^	
Boredom	−0.30^***^	−0.82^***^		−0.74^***^	−0.74^***^		−0.94^***^	−0.94^***^	
Anxiety	−0.15^***^	−0.49^***^		−0.52^***^	−0.52^***^		−0.46^***^	−0.46^***^	
Boredom			0.12^**^			0.15^**^			0.07^†^
Perceived academic control	−0.43^***^	−0.35^***^		−0.42^***^	−0.39^***^		−0.28^**^	−0.27^**^	
Anxiety	0.09^***^	0.30^***^		0.25^**^	0.25^**^		0.40^***^	0.40^***^	
Anxiety			0.30^***^			0.34^***^			0.30^***^
Perceived academic control	−0.64^***^	−0.55^***^		−0.72^***^	−0.59^***^		−0.66^***^	−0.55^***^	
**Indirect relation (tests of mediations)**	**Hypothesized model**			**Final partial invariant multi-group model**					
	**Total sample (*N* = 883)**			**Freshman sample (*N* = 597)**			**Second-year sample (*N* = 286)**		
	***b*** **(*****p*****)**	β **(*****p*****)**		***b*** **(*****p*****)**	β **(*****p*****)**		***b*** **(*****p*****)**	β **(*****p*****)**	
**Dropout intention**									
Perceived academic control via enjoyment	−0.06 (0.120)	−0.07 (0.116)		−0.06 (0.397)	−		−0.25 (0.068)	−	
Perceived academic control via boredom	0.05 (0.180)	0.06 (0.179)		0.01 (0.883)	−		0.25 (0.088)	−	
Perceived academic control via anxiety	−0.30^***^	−0.35^***^		−0.54^***^	−		−0.43^***^	−	
**Academic achievement**									
Perceived academic control via enjoyment	−0.02 (0.741)	−0.01 (0.742)		0.03 (0.316)	−		−0.09 (0.404)	−	
Perceived academic control via boredom	0.01 (0.893)	0.01 (0.893)		−0.01 (0.725)	−		0.07 (0.513)	−	
Perceived academic control via anxiety	−0.04 (0.518)	−0.03 (0.516)		−0.04 (0.327)	−		0.03 (0.360)	−	

*Mediation effects of academic emotions of the impact of perceived academic control on academic achievement. The independent variables of each dependent variable are listed indented below the construct. The estimates presented were derived from the final partial invariant model (refer to the model 7 in Table 5). MLR maximum likelihood parameter estimates*.

† *p < 0.10*,

* *p < 0.05*,

** *p < 0.01*,

*** *p < 0.001*.

As expected, perceived academic control related to all three academic emotions, particularly to anxiety. The more students felt in control the less they experienced anxiety. In contrast to the bivariate correlation, perceived academic control had no significant effect on dropout intention when controlling for the shared variance with the academic emotions. The feeling of control itself did not relate to low dropout intention when controlling for academic emotions. However, perceived academic control positively predicted academic achievement, similar to the bivariate correlations, while controlling for the shared covariance of academic emotions. High beliefs about personal control predicted high academic achievement. On the other hand, the moderate to strong correlations between all academic emotions and dropout intention reduced to a strong relationship between only anxiety and dropout intention in the SEM. Taking all three academic emotions and perceived academic control into account, only anxiety significantly related to dropout intention. The more students experienced anxiety the more likely they intended to drop out. Surprisingly, boredom and anxiety had no specific predictive effects on achievement when controlling for perceived academic control, contrary to the correlation results. Moreover, our model for the total sample showed a moderate relationship between the two steps of academic success, dropout intention and academic achievement, similar to the bivariate correlations. Undergraduate students with a strong intention to drop out tended to achieve poorly.

Finally, we analyzed the hypothesized mediations of academic emotions. Concerning dropout intention, the predictive effect of perceived academic control was fully mediated by anxiety^5^. Enjoyment and boredom did not show mediational effects. Undergraduate students with low levels of perceived academic control tended to experience strong anxiety toward studying, and as a result had higher intentions to dropout. Concerning academic achievement, the results showed no mediations, as only perceived academic control had a predictive effect on academic achievement. In addition to this mediation, the durations of study (e.g., first vs. second year) could have moderated these relationships. Therefore, we analyzed moderated mediations through multi-group structural equation analysis, but first we checked for measurement invariance.

### Research question 2–testing differences between freshman and second-year students

Table 5 displays the results of the hierarchical set of measurement invariance tests. We confirmed configural measurement invariance when we found the hypothesized model with free estimated factor loadings via sufficient fit indices in both subgroups separately (model 0) as well as in the configural structural equation model (unrestricted multi-group SEM, model 1). However, due to the weaker model fit and the significant chi-square difference test, model 2 (constrained factor loadings to be invariant across the two subgroups) did not hold up against the configural model (model 1). Therefore, we could not confirm weak measurement invariance. As results of analysis by Byrne et al. (1989), multi-group SEM analysis can continue under the condition of partial measurement invariance. We analyzed the residual covariance of all items one by one (model 3 and model 4) to identify factor loadings that should not be constrained to be equal in order to confirm partial weak measurement invariance (as recommend by Muthén and Muthén, 1998–2012; Byrne, 2012). We started these *post-hoc* analyses with the items that when not constrained suggested the greatest change of the chi-square value (model 3, item pac_1). We stopped these *post-hoc* analyses as the chi-square difference test became non-significant and the model fits were similar to the configural model again (model 4, items pac_1 and pac_2). Two factor loadings of perceived academic control differed across both student groups (“The more effort I put into my courses, the better I do in them” and “No matter what I do, I can't seem to do well in my courses”). We could not confirm full strong measurement invariance, as the chi-square difference test was significant when we constrained all intercepts and factor loadings to be equal across groups, with the exception of the two items of perceived academic control (model 5). Therefore, we again executed *post-hoc* analyses and found the intercept of one perceived academic control item (model 6) and one dropout intention item (model 7) were unequal across the two student groups (perceived academic control: “No matter what I do; I can't seem to do well in my courses” and dropout intention “I am likely to change my major”). The final measurement invariance model (model 7) confirmed partial strong measurement invariance with an adequate model fit. In this model, all factors loadings, besides pac_1 and pac_2, as well as all intercepts, besides pac_2 and dro_1, were constrained to be invariant between the two subgroups.

**Table 5:** **Structural invariance analysis: summary of model fit and χ^2^-Difference-Test-Statistics**.

**Invariance level**	**MLRχ^2^**	**χ^2^df**	**CFI**	**RMSEA**	**SRMR**	**Model Comparison**	ΔMLR χ^2^^*^	**Δdf**	***p***
**Configural model**									
(Model 0) Baseline group 1	267.60	136	0.95	0.04	0.04				
(Model 0) Baseline group 2	242.70	136	0.94	0.05	0.06				
(Model 1) Configural model	510.20	272	0.95	0.04	0.05				
**Factor loadings**									
(Model 2) All factor loadings invariant	549.03	290	0.94	0.05	0.08	1 vs. 2	38.12	18	0.004
(Model 3) All factor loadings invariant except for pac_1	540.50	289	0.94	0.04	0.08	3 vs. 2	6.35	1	0.012
						3 vs. 1	30.53	17	0.023
(Model 4) All factor loadings invariant except for pac_1 & pac_2	533.63	288	0.95	0.04	0.07	4 vs. 3	7.61	1	0.006
						4 vs. 1	24.50	16	0.079
**Intercepts**									
(Model 5) All intercepts invariant	611.20	301	0.93	0.05	0.07	5 vs. 4	82.67	13	<0.001
(Model 6) All intercepts invariant except for pac_2	573.35	300	0.94	0.05	0.07	6 vs. 5	43.40	1	<0.001
						6 vs. 4	41.32	12	<0.001
(Model 7) All intercepts invariant except for pac_2 & dro_1	547.59	299	0.94	0.04	0.07	7 vs. 6	20.34	1	<0.001
						7 vs. 4	13.11	11	0.286

* *MLR corrected values*.

*All χ^2^ were significant at p < 0.001*.

After establishing partial strong measurement invariance, we were able to compare the latent means of our study variables (Marsh and Grayson, 1994). Only the average level of perceived academic control, anxiety, and dropout intention differed between the two student groups. Compared to freshmen, second-year students reported lower anxiety (_Δ_*M* = −0.56, *p* < 0.001, *d* = −0.46) and lower dropout intention (_Δ_*M* = −0.67, *p* < 0.001, *d* = −0.47). Interestingly, second-year students also reported significantly lower perceived academic control compared to freshmen (_Δ_*M* = −0.33, *p* < 0.001, *d* = −0.32). Additionally, we found a significant mean difference for the observed variable of academic achievement across the two groups (*F* = 21.74, *p* < 0.001; *t*_(693)_ = 6.56, *p* < 0.001^6^). Specifically, freshmen had an average lower GPA than advanced students (_Δ_*M* = −0.29, *d* = 0.47). This means second-year students achieved better compared to students of the first academic year, irrespective of their study subject.

Turning now to invariances of the final path model across two different student groups, some structural paths significantly varied depending on the duration of study. After establishing partial strong measurement invariance, we used model 7 as a baseline model. Table 6 (upper part) displays the results of the individual invariance tests of the structural parameters in the model. Fifteen individual parameter invariance tests were conducted. Five path coefficients were found to be different across student groups. Therefore, we fixed the remaining 10 invariant path coefficients to be equal across student groups in the final multi-group model (model 8). This final model displays the structural differences of first-year students and second-year students, which had an adequate model fit.

**Table 6:** **SEM model fit statistics and results of direct and indirect structural invariance tests**.

**Individual path coefficient constrained**	**MLRχ^2^**	**χ^2^df**	**ΔMLR χ^2^**	**ΔMLR χ^2^*p* value**	**ΔMLR χ^2^ test (α = 0.05)**	**CFI**	**RMSEA**	**SRMR**
**Dropout intention**								
Perceived academic control	552.12	300	6.55	0.010	Non-invariant	0.94	0.04	0.07
Enjoyment	550.24	300	3.96	0.047	Non-invariant	0.94	0.04	0.07
Boredom	551.17	300	10.68	0.001	Non-invariant	0.94	0.04	0.07
Anxiety	547.42	300	0.22	0.639	Invariant	0.94	0.04	0.07
Academic achievement	548.83	300	1.13	0.288	Invariant	0.94	0.04	0.07
**Academic achievement**								
Perceived academic control	548.51	300	0.59	0.442	Invariant	0.94	0.04	0.07
Enjoyment	550.20	300	0.70	0.403	Invariant	0.94	0.04	0.07
Boredom	548.81	300	1.03	0.310	Invariant	0.94	0.04	0.07
Anxiety	551.31	300	184.42	<0.001	Non-invariant	0.94	0.04	0.07
**Academic emotion enjoyment**								
Perceived academic control	548.06	300	0.63	0.427	Invariant	0.94	0.04	0.07
Boredom	558.44	300	12.57	<0.001	Non-invariant	0.94	0.04	0.07
Anxiety	547.25	300	0.37	0.543	Invariant	0.94	0.04	0.07
**Academic emotion boredom**								
Perceived academic control	549.52	300	1.93	0.165	Invariant	0.94	0.04	0.07
Anxiety	548.96	300	1.48	0.224	Invariant	0.94	0.04	0.07
**Academic emotion anxiety**								
Perceived academic control	547.20	300	0.27	0.603	Invariant	0.94	0.04	0.07
**(model 8) Final model^a^**								
10 invariant path coefficients constrained	562.13	309	14.79	0.140	Invariant	0.94	0.04	0.07
**Indirect effects difference test**	**MLRχ^2^**	**χ^2^df**	**difference *z*-score**	***z*-test *p*-value**	***z*-test (α = 0.05)**	**CFI**	**RMSEA**	**SRMR**
**Dropout intention**								
Perceived academic control (via enjoyment)	547.59	299	1.189	0.235	Invariant	0.94	0.04	0.07
Perceived academic control (via boredom)	547.59	299	−1.422	0.155	Invariant	0.94	0.04	0.07
Perceived academic control (via anxiety)	547.59	299	−0.651	0.515	Invariant	0.94	0.04	0.07
**Academic achievement**								
Perceived academic control (via enjoyment)	547.59	299	−1.10	0.274	Invariant	0.94	0.04	0.07
Perceived academic control (via boredom)	547.59	299	0.74	0.460	Invariant	0.94	0.04	0.07
Perceived academic control (via anxiety)	547.59	299	1.345	0.179	Invariant	0.94	0.04	0.07

*The estimates presented were derived from the final partial invariant model (refer to model 7 in Table 5). MLR corrected values*.

a *10 of 15 path coefficients found invariant in the chi-square difference tests were constrained to be equal across groups*.

**Figure 2:**
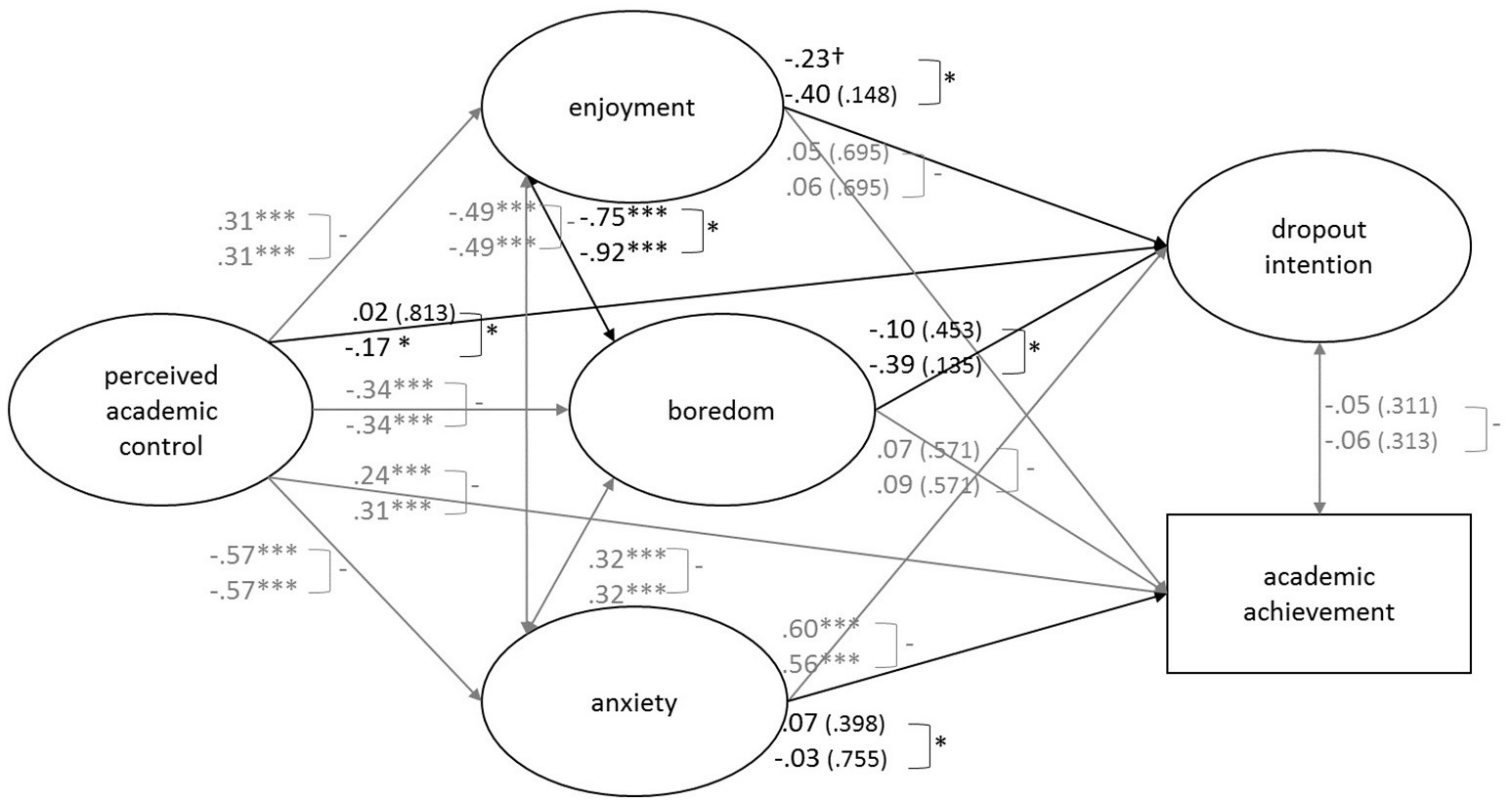
**Structural paths of the final multi-group SEM results**. The numbers refer to standardized MLR maximum likelihood corrected parameter estimates. Upper numbers refer to group of freshman students. Lower numbers refer to group of second-year students. 10 of 15 path coefficients found invariant in the chi-square difference tests were constrained to be equaled across groups (gray clip with −). The estimates presented were derived from the final partial invariant model (refer to model 8, see Table 6), if non-significance: *p*-value within the parenthesis, if marginal significance: †*p* < 0.10, significance level: ^*^p < 0.05, ^***^p < 0.001.

Figure 2 displays the results of the final multi-group model (model 8). Concerning the direct relationships with dropout intention, only anxiety was invariant across the students groups (Table 4, second column). Independent of the students' year at university, experiences of high anxiety strongly related to high intention to drop out. Alternatively, the predictive effect of perceived academic control, enjoyment, and boredom on dropout intention differed depending on the duration of study. We found expected pathways for students at the second academic year, however, the impact of perceived academic control on dropout intention marginally missed conventional levels of statistical significance (*p* = 0.059). Surprisingly, these relationships with dropout intention were non-significant for freshmen. Concerning the direct relations with achievement, only anxiety was different across the two student subgroups, but with non-significant impact. Due to high standard errors for the paths of second-year students, the moderate to strong path estimates of enjoyment (S.E. = 0.71) and boredom (S.E. = 0.70) were non-significant (Table 4, third column). Concerning the relationship of academic achievement and dropout intention, the two components of academic success were not related for either of the two students groups (freshmen vs. second-year students) and their relation was group-invariant.

We additionally tested the invariance of our hypothesized mediations of academic emotions (Table 6, lower part). We found no moderated mediation for the two student groups. The strong mediational effect of anxiety on the impact of perceived academic control on dropout intention was not moderated by the duration of study. When students experienced low anxiety, their feeling of control had a negative impact on their dropout intention, independent of whether they were freshmen or second-year students.

## Discussion

For a better understanding of student success in terms of dropout intention and academic achievement, the present study focused on the critical first year of university. We examined undergraduate students' perceived academic control and academic emotions using a self-reported survey completed in a typical learning situation to answer two research questions. A summary of the results and a discussion of their implications are presented below.

### Research question 1–how perceived academic control and academic emotions predict undergraduate students' academic success

As we found mostly expected bivariate correlations, we confirmed our hypothesized relationships with their shared variance accounted for through SEM. The model results emphasized the importance of perceived academic control and anxiety for undergraduate students' academic success.

As stated, the correlations confirmed previous findings (e.g., Perry et al., 2001; Stupnisky et al., 2013) concerning the relationships between perceived academic control with enjoyment, boredom, and anxiety. Our model also confirms the importance of perceived academic control for undergraduate students' academic success (e.g., Stupnisky et al., 2008) based on the expected positive predictive effect of perceived academic control on achievement even when controlling for academic emotions. In our model we did not, however, find a direct relationships between perceived academic control and dropout intention. Interestingly, the direct medium negative correlation between perceived academic control and dropout intention (compared to Ruthig's AERA presentation 2002 as cited in Perry et al., 2005a, p. 384) became non-significant as our model revealed a full mediation through anxiety. This broadened the findings from Ruthig et al. (2008), who found anxiety to moderate the predictive effect of perceived academic control on academic achievement. This result of the present study also emphasizes the importance of anxiety for undergraduate students. The full mediation could be due to individual significance. Undergraduate students just recently chose to study as well as their major and therefore appreciate university highly. Anxiety occurs when learning situations or their outcomes are highly valued with low perceived control (Pekrun, 2006). Thus, anxiety has a strong relation with the intention to drop out of university, even when controlling for perceived academic control. Furthermore, the model explains nearly 50 percent of the dropout intention variance, which again underlines the importance of perceived academic control and anxiety.

In our models, we could not find specific relationships between academic emotions and achievement, unlike the results of prior research (e.g., Pekrun et al., 2011; Putwain et al., 2013). The small negative relation of the negative emotions with achievement became non-significant when we estimated their predictive effects excluding shared variance with perceived academic control and enjoyment. Therefore, we cannot fully confirm all expected influences of academic emotions, unlike Ruthig et al. (2008). Surprisingly, only perceived academic control had a predictive effect on the academic achievement component of academic success. One possible explanation could be feedback loops as postulated in the control value theory (Pekrun, 2006). Academic emotions can influence perceived academic control and therefore academic achievement. Furthermore, we obtained academic achievement at the end of the semester, unlike previous studies that obtained GPA simultaneously (e.g., Pekrun et al., 2010; Mega et al., 2014) or prior to the study (Pekrun et al., 2011). This time-delayed measurement of achievement possibly showed the importance of time span for the predictive effects of academic emotions. Moreover, the missing effects of the emotions on achievement could be the results of their operationalization. As the external validation study of the AEQ showed, course-related emotions have a weaker relationships with achievement compared to learning- and test-related emotions (Pekrun et al., 2011). We measured course-related emotions within a subject-critical lecture, as opposed to test-related emotions right after an exam. Other crucial factors may have intervened to result in the non-significant effects of the academic emotions: cognitive resources (Pekrun, 2006), self-regulated learning strategy usage (Pekrun et al., 2002), or goal orientations (as shown for boredom and anxiety; Pekrun et al., 2009). Furthermore, the lack of relationships among academic emotions with academic achievement in our SEM could be due to our interdisciplinary sample that consists of several different disciplines offered by a German STEM university. STEM students are analytically minded and might therefore rarely reflect upon and cope with their emotions while learning (compared to more self-reflexive students such as from psychology). This expands the generalization of our findings, as previous research often focused on psychology students or students of introductory courses and undecided majors (e.g., Perry et al., 2005b; Ruthig et al., 2009; Stupnisky et al., 2013).

Additionally, in our model we found a small relationship between dropout intention and academic achievement, as the correlations already suggested and in line with prior research (e.g., Pascarella and Terenzini, 2005; Allen et al., 2008). Undergraduate students who intend to dropout at the beginning of the semester are more likely to achieve poorly at the end of the semester, independent of their level of perceived academic control or experienced academic emotions at the beginning of the semester. This small relationship between the two components of academic success was not present, however, when we compared the two student cohorts.

### Research question 2–how the prediction of academic success differ for freshman students vs. second-year students

The present study compared students from two different cohorts, namely freshman students and second-year students, through multi-group analysis. We found good support for our hypothesized multi-group model: The measurement models were partially invariant and most path coefficients were invariant across the two student groups. Consequently, we validated our model for the total sample for freshman students and second-year students. Only two factor loadings of the perceived academic control latent variable differed between the sub-groups, which could represent the different students' frame of reference for their perceptions (secondary school vs. higher education).

In line with Pekrun (2006) and Frenzel et al. (2007), only the latent means of perceived academic control, dropout intention, and anxiety differed between both groups. As expected, second-year students perceived less academic control (Stupnisky et al., 2012), experienced less anxiety (Ranellucci et al., 2015) and had lower dropout intention (Bean, 1985) compared to freshmen. One possible explanation for the reduction in perceived academic control over the first academic year could be failure experiences. Students tend to decrease their perceived academic control after failure experiences (Hall, 2008). We collected data from freshman students in the first semester before their first university exam. Thus, these students may not have experienced much failure yet, which leads to higher perceived academic control. Additionally, the advanced sample had significantly better grades, with low variance and skewness. Perhaps this sample contained more high-achieving students or the low achieving students had dropped out, while the freshman sample contained a wider variance. Beyond failure experiences, other explanations could be the transparency of educational requirements. Students who know what to expect in the lecture, what the educational goals are, and how they will be tested should have higher perceived academic control (Stupnisky et al., 2007). However, second-year students' expectancy about goals and tests may have been repeatedly unmet, or they may have missed critical information about the tests as teachers or instructors might failed to provide these as clearly in second year courses, leading to lower perceived control.

Concerning the invariances of the final path model across the two student groups, few structural paths significantly varied depending on students' year in university. Freshman students differed slightly from second-year students, not only in their level of perceived academic control, academic emotions and academic success, but also in some relationships between these crucial variables. In general, the students mainly differed concerning their dropout intention and factors influencing this intention to drop out of university. This difference could have occurred due to the special sample of second-year students. All participants in the second group already successfully completed their first academic year and therefore the sample did not include students who dropped out. This could also explain the lower anxiety and dropout intention mean level reports. Compared to the freshmen cohort, the path results of the second-year students are more in line with prior research, as perceived academic control now related to dropout intention directly (Ruthig's AERA presentation 2002 as cited in Perry et al., 2005a, p. 384). The more second-year students perceived control over their academic outcomes, the less they intended to drop out, independent of their academic emotions and achievement. Additionally, the predictive effect of perceived academic control on the achievement was stronger for second-year students compared to freshmen. These results demonstrate the importance of perceived academic control for the combined components of academic success in the second academic year. Furthermore, the results show the significance of enjoyment and boredom in the second academic year, due to the strong relationships to dropout intention, compared to the freshmen group where we found no relationship. Consistent with prior research (Ruthig et al., 2007), we found that enjoyment was a strong protective factor against dropout intention for second-year students.

Concerning achievement, the correlations differed between the two subgroups regarding enjoyment, only small relations for the freshmen group, and anxiety, only small relations for the second-year student group. Considering their specific predictive effects, enjoyment and boredom had medium to strong relationships with achievement for second-year students compared to freshmen, however were non-significant due to high standard errors. These could be due to multicollinearity between enjoyment and boredom, which are highly correlated (*r* = −0.80, Table 2), or due to the heterogeneous advanced sample, consisting of many different majors.

Finally, the results showed a small relationship between the two components of academic success for the total sample, which vanished when the two subgroups were compared. We found no specific relationship between dropout intention at the beginning of the semester with achievement at the end of the semester, when taking perceived academic control and academic emotions into account. These results replicate Bean's (1985), who found a significant relation between dropout intention and academic achievement only when considering all undergraduate students compared to the undergraduate cohorts separately. These path results are contrary to the correlations, where the freshmen group showed no relationship between the components of academic success, but the second-year student group showed a medium relation. This again underlines the importance of perceived academic control and anxiety, particularly for second-year students, as the specific impact of perceived academic control and anxiety reduces the dropout-achievement relation. It looks like freshman students tend to drop out at the beginning of their studies independent of their achievement later on. This suggests that dropout intention is specifically important for freshman students as even high achievers might have intentions to drop out. Moreover, the model for second-year students showed higher explained variance of achievement as well as dropout intention, compared to the model for freshmen. It seems the hypothesized model applies most closely for experienced students.

### Strengths and limitations

The results must be viewed within the strengths and limitations of the study. A major strength of the present study is that it focused on the critical first academic year as it influences overall academic success (Credé and Niehorster, 2012). Another contribution of our study is its extension of the limited research on dropout intention (Bean, 1982). The present study also improves upon prior higher education research that typically focuses on negative emotions (similar to Ruthig et al., 2008). Several methodological strengths of the present study include a field-based design within the natural environment, high ecological validity through a wide range of study subjects, time-delayed measurement of achievement, multi-item and established scales to create latent variables, and SEM to account for measurement errors and shared variance.

Although the study provided insights into the relationships between perceived academic control, academic emotions, and undergraduate students' academic success, there were limitations. One limitation may be that only GPA was measured following a time lag; therefore, causality interpretations are limited to the prediction of achievement. However, it is an important first step to understand the relations between perceived academic control, academic emotions, and academic success as well as the differences between freshmen and second-year students. Another limitation might be our operationalization of dropout intention. It included the likelihood to change majors, which could be understood as transition instead of dropout. However, this distinction is contextual as changing to another major represents a dropout for a particular major, but transition for the head of the university overall. Another limitation concerns the selection bias of the sample, as the students needed to attend the lecture to participate in the study. Considering the second-year students, the reason for the rather low response rates may be multifaceted: they have participated in many other student surveys, have more complex timetables, practice more self-regulated learning behavior and therefore it could be unnecessary to attend the lecture or participate in the survey due to competing demands. The response rates of the second-year students should not have influenced our study results. However, the generalization of the results could be limited to high-motivated students, which regularly attend lectures. Finally, another limitation of the current study concerns the GPA release form. Students who did not allow us to obtain their GPA might be low-achievers, but many other explanations also could have occurred. In general, this selective sample could have biased the results to create larger effects or limited the variance to undermine our results.

## Conclusion and implications for the higher education

One major contribution of the present study is that it extends research on the predictive effects of perceived academic control and academic emotions on academic success by examining the intention to drop out of university as an additional outcome. Prospective research should test the model against actual dropout, despite its methodological challenges (Allen et al., 2008). Another primary finding of the present study is the importance of perceived academic control for academic success, specifically within the first year. An extension to the current study would be to examine the relationships of perceived academic control to other predictors for undergraduate students' academic success, such as achievement motivation (c.f. Hall et al., 2006; Daniels et al., 2014). Freshmen might have different goal orientations or different intrinsic motivation compared to second-year students due to their expectations and lack of experience. Due to multicollinearity, however, future research should analyze the relationships separately for each predictor in multiple studies. Furthermore, future research should include value in order to verify the full control-value theory (Pekrun, 2006) for freshman university students. However, the German freshmen chose their specific major for the whole Bachelor degree, which leads to high subjective value, and it is difficult to increase through institutional activities.

Our results highlight the possible protective effect of enjoyment for dropout intention, in addition to the avoidance of anxiety (similar to Ruthig et al., 2008), which should be established in subsequent research. Moreover, our results show specific characteristics of undergraduate students at certain times during their first two academic years, as we found slightly different results for incoming students compared to advanced students. Therefore, future research should analyze these characteristics in more detail and combine adjustment research with research focused on enhancing academic success. Moreover, researchers should also aim to reanalyze these interrelations with a complete cohort of university students, as our study included a selected sample. Moreover, possible reasons for dropout surrounding the GPA release form should be further investigated. However, due to the practical difficulties of a field study in general, we still achieved a high response rate for freshman students and a moderately acceptable response rate for second-year students. Finally, the present study illuminates the importance of perceived academic control and anxiety for academic success within the first two academic years. It would be interesting to follow up on the intraindividual development of these important predictors, in addition to this interindividual comparison. With a longitudinal design, subsequent researchers could identify possible feedback loops of the mediational effects of academic emotions on the relationship between perceived academic control and academic success, as well as establish the different impacts of course-, learning-, and test-related emotions. Moreover, further longitudinal studies can analyze the causality of perceived academic control on dropout intention via academic emotions.

Our results also have practical implications for higher education institutions. They show the importance of perceived academic control and academic emotions on academic success. Thus, instructors should support students' perceived academic control and positive academic emotions in order to reduce dropout intention and increase achievement. As perceived academic control linked to dropout intention in the current study, perceived academic control enhancement interventions (such as attributional retraining) would thus be logical techniques for universities to implement. Universities should offer attributional retraining early in the academic year (Perry et al., 2005a) or add the principles of it to freshman-level course (Ruthig et al., 2009). This would support students to reframe the way they think about failure as well as perceived control by encouraging them to assume responsibility and adopt a “can-do” attitude (Haynes et al., 2009). Likewise, the instructor can increase perceived academic control through information or discussions about good approaches to prepare for tests. This information enables students to anticipate academic outcomes and make the appraisal of achievement more transparent (Stupnisky et al., 2007; Ruthig et al., 2009). Similarly, instructors can create a high-control environment in their course, for example through clear course structure, transparent grading criteria, or being readily available for questions (Stupnisky et al., 2008) as well as enhancing individualistic and cooperative goal structures, adequate achievement expectations, or avoidance of cumulative failure feedback (Pekrun, 2006). Similarly, Tinto (2010) recommended early assessment and feedback to increase the predictability of the course demands, and therefor increase perceived academic control to reduce dropout intentions. Additionally, professors should pay attention to the students' emotions (Pekrun et al., 2011) in order to reduce dropout intentions. Universities could offer coaching regarding emotion regulation, as recommend by Hall and Goetz (2013). Moreover, our results emphasize the importance of the first months at university, as many freshman-supporting programs within higher education already assumed.

In conclusion, our findings provide new insights into the experiences of university students during their critical first two academic years and possible predictors of academic success, with respect to both reducing dropout intention and increasing academic achievement. Perceived academic control and academic emotions, specifically anxiety, do matter to undergraduate students. Luckily, as noted above, they can be supported by higher education institutions.

## Ethics statement

This study was exempt from an ethic committee approval due to the recommendations of the German Research Association: All subjects were in no risk out of physical or emotional pressure, we fully informed all subjects about the goals and process of this study and none of the subjects were patients, minors or persons with disabilities.

## Author contributions

LR drafted the work, which was revised critically by UN, RS, and TS. LR and UN contributed to all steps of the work. RS and TS contributed to the interpretation of the data for the work. All authors approve of the final version of the manuscript and agree to be accountable for all aspects of the work in ensuring that questions related to the accuracy or integrity of any part of the work are appropriately investigated and resolved.

### Conflict of interest statement

The authors declare that the research was conducted in the absence of any commercial or financial relationships that could be construed as a potential conflict of interest.

## Footnotes

^1^Perceived academic control is an independent latent variable that captures students' beliefs about their personal influence and control over their academic outcomes. Academic emotions enjoyment, boredom, and anxiety are represented as latent variables and express students' enjoyment of learning, boredom, and anxiety (respectively) concerning their studies and learning activities within the first two academic years. High values indicated that students feel more enjoyment, boredom or anxiety. Dropout intention is a dependent latent variable. High values on this variable indicated high likelihood of dropout. Academic achievement is a dependent observed variable (low GPA indicated low academic achievement).

^2^We tested for significant differences in the study variables between students who did vs. did not consent to their grades being collected. Only perceived academic control differed slightly. Students who did not consent to their grades being collected reported a lower perceived academic control (Bonferroni adj., *t* (454) = 3.22, *p* = 0.001, _Δ_*M* = −0.14).

^3^A computation with non-centered GPA measures showed very similar results for our hypothesized model (comparable fit indexes, structural pathways and significances).

^4^Some participants dropped out between the two points of data collection. The missing data contains de-registrations from 31 freshmen (5.19% of the freshman group) as well as 5 s-year students (1.77% of the second-year group). Due to various reasons (e.g., no comprehensive survey, no GPA release form from all participants, delay within the institutional records etc.), these dropout rates do not represent the actual dropout rates of these cohorts.

^5^We confirmed the significance of these mediational effects via bootstrapping. The standard bootstrap estimates were obtained based on 10,000 bootstrap samples, as recommend by Mallinckrodt et al. (2006).

^6^We used the parametric t-test, because the graphical check suggested normality distributions. However, the non-parametric one sample Kolmogorov-Smirnov test also would have been significant.
