# Supplementary material for: Perceived Academic Control and Academic Emotions Predict Undergraduate University Student Success: Examining Effects on Dropout Intention and Achievement
Source: Front Psychol. 2017 Mar 7;8:243. doi: 10.3389/fpsyg.2017.00243 (PMC5339277; doi:10.3389/fpsyg.2017.00243)
Supplement: Supplementary file 1 [file DataSheet1.docx]

## Appendix

#### Mplus Syntax SEM (total sample)

**Variable:**

NAMES ARE …. ;

MISSING ARE all (9999);

USEVARIABLES ARE … ;

**Analysis:**

ESTIMATOR = MLR;

TYPE IS general;

**Model:**

jo BY jo_1 jo_2 jo_3; !enjoyment

bo BY bo_1 bo_2 bo_3; !boredom

ax BY ax_1 ax_2 ax_3; !anxiety

pac BY pac_1 pac_2 pac_3 pac_4 pac_5 pac_6; !perceived academic control

drop BY dro_1 do_2 dro_3; !dropout intention

bo_3 WITH bo_2; !post-hoc analysis

ax_2 WITH ax_1;

gpa ON pac jo bo ax ; !hypothesized model

drop ON ax pac jo bo ;

jo bo ax ON pac ;

jo WITH bo;

jo WITH ax;

bo WITH ax;

gpa WITH drop;

MODEL INDIRECT:

drop IND pac;

gpa IND pac;

#### Mplus Syntax SEM (multi-group)

**Variable**:

NAMES ARE …. ;

MISSING ARE all (9999);

USEVARIABLES ARE … ;

GROUPING IS cur_sem ( 1= freshman group 3= second-year group);

**Analysis**:

ESTIMATOR = MLR;

TYPE IS general;

**Model**:

drop BY dro_1* dro_2 dro_3; !model freshman group

drop@1;

jo BY jo_1* jo_2 jo_3;

jo@1;

bo BY bo_1* bo_2 bo_3;

bo@1;

ax BY ax_1* ax_2 ax_3;

ax@1;

pac BY pac_1* pac_2 pac_3 pac_4 pac_5 pac_6;

pac@1;

bo_3 WITH bo_2;

ax_2 WITH ax_1;

gpa ON pac jo bo ax ; !hypothesized model

drop ON ax pac jo bo ;

jo bo ax ON pac ;

jo WITH bo;

jo WITH ax;

bo WITH ax;

gpa WITH drop;

MODEL SECOND-YEAR GROUP:

pac BY pac_1 pac_2;

[ PAC_2 ];

[ dro_1 ];

#### Mplus Syntax SEM (moderated mediation)

**Variable**:

NAMES ARE …. ;

MISSING ARE all (9999);

USEVARIABLES ARE … ;

GROUPING IS cur_sem ( 1= freshman group 3= second-year group);

**Analysis**:

ESTIMATOR = MLR;

TYPE IS general;

**Model**:

drop BY dro_1* dro_2 dro_3;

drop@1;

jo BY jo_1* jo_2 jo_3;

jo@1;

bo BY bo_1* bo_2 bo_3;

bo@1;

ax BY ax_1* ax_2 ax_3;

ax@1;

pac BY pac_1* pac_2 pac_3 pac_4 pac_5 pac_6;

pac@1;

bo_3 WITH bo_2;

ax_2 WITH ax_1;

gpa ON pac (1)

jo (3)

bo (4)

ax ;

drop ON ax (5)

pac jo bo ;

jo ON pac (6);

bo ON pac (8);

ax ON pac (10);

jo WITH bo;

jo WITH ax (7);

bo WITH ax (9);

gpa WITH drop (2);

MODEL FRESHMAN GROUP

drop ON ax (p1a);

ax ON pac (p2a);

MODEL SECOND_YEAR GROUP

pac BY pac_1 pac_2;

[ PAC_2 ];

[ dro_1 ];

drop ON ax (p1b);

ax ON pac (p2b);

MODEL CONSTRAINT:

NEW (ind_1 ind_2 diff);

ind_1 = p1a*p2a; !mediation

ind_2 = p1b*p2b;

diff = ind_1-ind_2; !moderated mediation

#### Summary of the variables

| Item | M | SD |
| --- | --- | --- |
| Perceived Academic Control | | |
| 1. The more effort I put into my study, the better I do in it. | 4.02 | 0.84 |
| 2. No matter what I do, I can’t seem to do well in my courses. (R) | 2.22 | 0.96 |
| 3. I see myself as largely responsible for my academic performance. | 4.37 | 0.71 |
| 4. How well I do in my exams at university is often the ‘luck of the draw.’ (R) | 2.48 | 0.96 |
| 5. There is little I can do about my performance in university. (R) | 1.88 | 0.82 |
| 6. My grades are basically determined by things beyond my control and there is little I can do to change that. (R) | 1.88 | 0.87 |
| Enjoyment | | |
| 7. I get excited about going to my courses. | 3.88 | 0.80 |
| 8. I enjoy being in my courses. | 3.78 | 0.71 |
| 9. My enjoyment of my courses makes me want to participate. | 3.30 | 0.86 |
| Boredom | | |
| 10. I find my courses fairly dull. | 2.30 | 0.83 |
| 11. I get so bored I have problems staying alert. | 2.09 | 1.02 |
| 12. Because I get bored my mind begins to wander. | 2.90 | 1.17 |
| Anxiety | | |
| 13. Thinking about my courses makes me feel uneasy. | 2.21 | 1.03 |
| 14. When I think about my courses, I get queasy. | 1.78 | 0.94 |
| 15. Because I’m so nervous I would rather skip my courses. | 1.60 | 0.94 |
| Dropout Intention | | |
| 16. I am likely to change your major. | 1.68 | 0.70 |
| 17. I am likely to leave university temporarily | 1.64 | 0.65 |
| 18. I am likely to leave university permanently. | 1.54 | 0.62 |
